# Supplementary material for: In silico design of a T-cell epitope vaccine candidate for parasitic helminth infection
Source: PLoS Pathog. 2020 Mar 23;16(3):e1008243. doi: 10.1371/journal.ppat.1008243 (PMC7117776; doi:10.1371/journal.ppat.1008243)
Supplement: S1 Fig — Left: Chromatogram of the VLP recombinant proteins HBc-Ag (A), HBc-CBD1243-1259 (B), HBc-CBD241-257 (C), HBc-CLSP143-158, (D), and HBc-CLSP398-416 (E) purified using SEC. Separation was carried out on a Superose 6, 10/300 GL (GE Healthcare), at a flow rate of 0.2 ml/min with 100 mM Tris-HCl, 150 mM NaCl, 1 mM EDTAatpH 8. VLP recombinant proteins were eluted in 0.5 ml fraction and a sample of SEC the elution peak was visualised by SDS-PAGE. Vertical red lines indicate the elution volume of mass standards, from left to right: 2MDa, 670kDa. Right: Purification visualised by SDS-PAGE (10% gel) analysis stained with Coomassie blue. M: protein marker (Precision Plus protein standard, unstained, Bio-Rad); Lane 1: VLP recombinant protein after Strep-Tag affinity purification using the StrepTrap HP, and Lane 2: VLP recombinant protein after SEC purification. The arrow represents VLP recombinant protein monomer with an expected molecular mass of ~22 kDa. (PPTX) [file ppat.1008243.s001.pptx]

## Slide 1
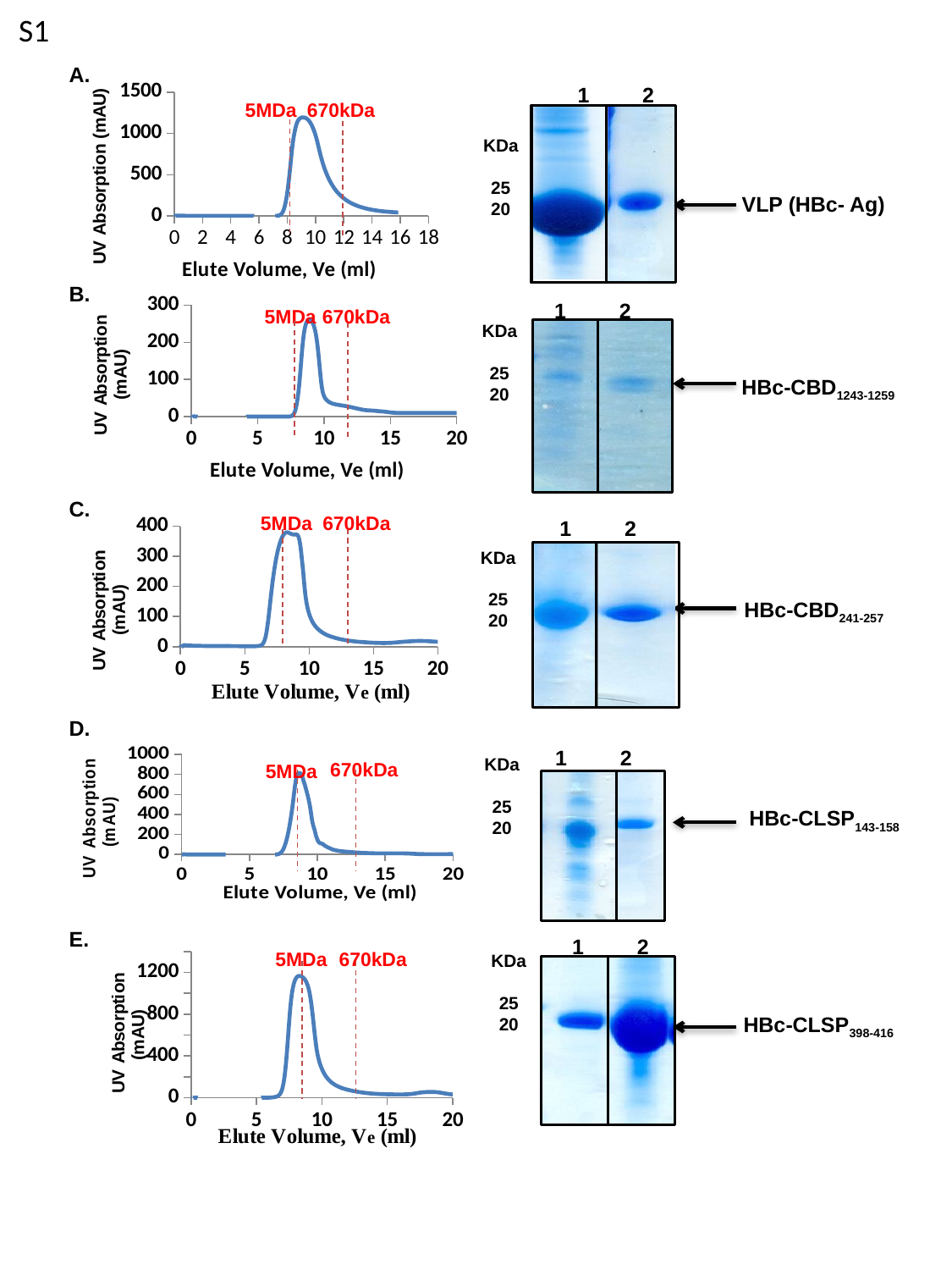

S1
A.
1 2
### Chart
| Category | |
|---|---|5MDa
670kDa
KDa
25
20
 VLP (HBc- Ag)
B.
### Chart
| Category | |
|---|---|1 2
5MDa
670kDa
KDa
25
20
HBc-CBD1243-1259
C.
5MDa
670kDa
### Chart
| Category | mAU |
|---|---|1 2
KDa
25
20
HBc-CBD241-257
D.
KDa
25
20
1 2
### Chart
| Category | | | |
|---|---|---|---|670kDa
5MDa
HBc-CLSP143-158
### Chart
| Category | mAU |
|---|---|E.
1 2
KDa
25
20
670kDa
5MDa
HBc-CLSP398-416
