# Supplementary figures and images for: In silico design of a T-cell epitope vaccine candidate for parasitic helminth infection
Source: PLoS Pathog. 2020 Mar 23;16(3):e1008243. doi: 10.1371/journal.ppat.1008243 (PMC7117776; doi:10.1371/journal.ppat.1008243)

## Slide 1
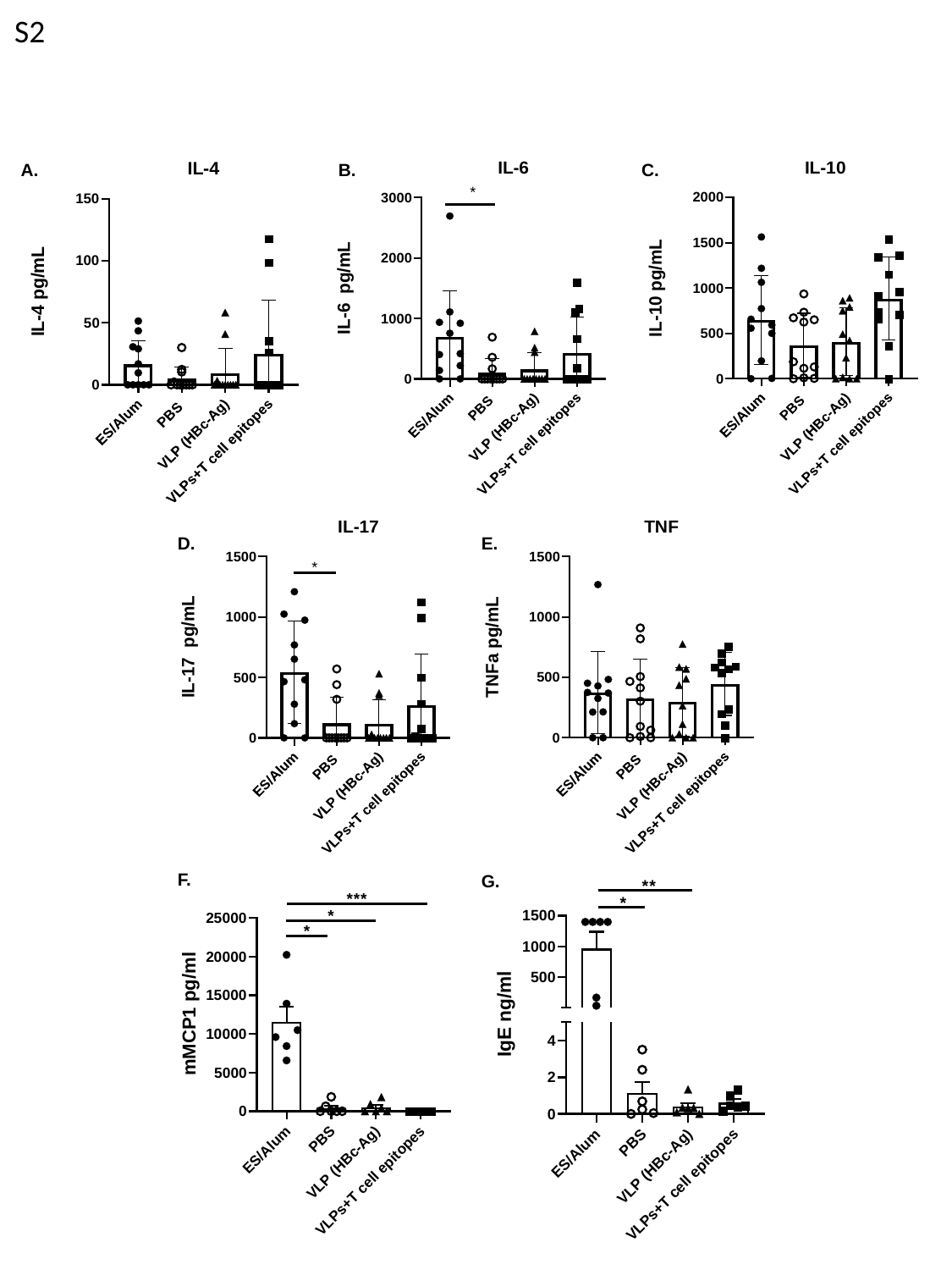

S2
A.
B.
C.
D.
E.
F.
G.

Supplement: S2 Fig — (A-E) Cytokine productions by mesenteric lymph node cells in mice vaccinated with VLPs+T-cell epitopes and control mice. (A) MLNs from mice vaccinated with 50 μg of pre-mixed of VLPs+T-cell epitopes (HBc-CBD1243-1259, HBc-CBD241-257, HBc-CLSP143-158 and HBc-CLSP398-416), 50 μg of VLP (HBc-Ag), 50 μg ES/Alum, or PBS on day -20 and boosted on day -10 were stimulated at 5x10^6^/ml with 50 μg/ml T. muris ES. After 48 hours of stimulation, cell culture supernatants were harvested and assayed by cytometric bead array for IL-4 (A), IL-6 (B), IL-10 (C), IL-17 (D), and TNF-α (E) production. Results are shown as mean ± SEM. n = 11 mice per group. The results presented are from two separated experiment pooled together. (F) Serum mMCPT-1 levels assayed at a 1:100 dilution utilising a mouse mMCPT-1 ELISA kit (Invitrogen) according to manufacturer’s instructions. (G) Day 14 p.i. sera were titrated against purified anti-mouse IgE to assess serum IgE antibody levels in VLPs+T-cell epitopes, VLP (HBc-Ag), PBS and in ES/Alum vaccinated mice by ELISA (reading at 405 nm, with reference of 570nm subtracted). Statistical analyses were carried out using the Kruskal-Wallis test (multiple comparisons). Significant differences between groups are represented by (*P≤0.05, **P≤0.01, ***P≤0.001) with a line. This experiment was repeated two times, and the ELISA results shown here are representative of the two experiments. (PPTX) [file ppat.1008243.s002.pptx]
