# Supplementary material for: In silico design of a T-cell epitope vaccine candidate for parasitic helminth infection
Source: PLoS Pathog. 2020 Mar 23;16(3):e1008243. doi: 10.1371/journal.ppat.1008243 (PMC7117776; doi:10.1371/journal.ppat.1008243)
Supplement: S1 Table — (DOCX) [file ppat.1008243.s003.docx]

S1 Table. List of Keywords used to screen for MHC I and II *in silico* prediction tools.

| Keywords |
| --- |
| Computational vaccinology |
| *In silico* vaccine |
| Peptide-based vaccine |
| T cell epitope vaccine |
| Epitope prediction algorithms |
| Binding affinity prediction |
| Bioinformatics prediction tools |
| Immunoinformatics prediction tools |
| *In silico* peptide docking assay |
| *In silico* tools for vaccine design |
| *In silico* epitope prediction |
| *In silico* design of epitope-based vaccines |
| T cell epitope identification |
| T cell epitope prediction |
| Prediction of CTL epitopes |
| T cell epitope prediction methods |
| T cell epitope prediction tools |
| Application of Epitope Driven Vaccine Design |
| Major histocompatibility complex prediction |
| MHC prediction |
| MHC prediction methods |
| MHC class II epitope prediction |
| MHC class I epitope prediction |
| MHC class II epitope prediction tools |
| MHC class I epitope prediction tools |
| MHC binding prediction |
| MHC class I binding prediction |
| MHC class II binding prediction |
| Prediction of MHC peptide binding |
| Identification of MHC binding motifs |
| Epitope prediction software |
| T cell epitope database |
| Databases for T cell epitopes |
| MHC database |
| Major histocompatibility complex database |
| Database for MHC ligands and peptide motifs |
| Epitope Analysis |
