# Supplementary material for: In silico design of a T-cell epitope vaccine candidate for parasitic helminth infection
Source: PLoS Pathog. 2020 Mar 23;16(3):e1008243. doi: 10.1371/journal.ppat.1008243 (PMC7117776; doi:10.1371/journal.ppat.1008243)
Supplement: S2 Table — (DOCX) [file ppat.1008243.s004.docx]

S2 Table. List of MHC class I and II and HLA binders peptides *in silico* prediction tools.

| **Prediction tool** | **Website** | **Prediction Method** | **MHC Class** |
| --- | --- | --- | --- |
| **BIMAS** | <http://www-bimas.cit.nih.gov/molbio/hla_bind/> | QM | I |
| **BJTEpitope** | <http://www.biosun.org.cn/bjtepitope/> | Naïve Bayes | I |
| **CombiPRED** | <http://www.vaccinedesign.com/> | A matrix-based tool that combines nHLAPred, BIMAS and SYFPEITHI | I |
| **CTLPred** | <http://crdd.osdd.net/raghava/ctlpred/> | QM, ANN and SVM | I |
| **EpiJen** | <http://www.ddg-pharmfac.net/epijen/EpiJen/EpiJen.htm> | Multistep algorithm (Proteasomal cleavage, TAP binders and MHC I binding) | I |
| **Expitope** | <http://webclu.bio.wzw.tum.de/expitope/> | NetChop, NetMHC and TAP affinity binding | I |
| **HLA Binding**  **MHC I peptide energy binding predictor** | <http://boson.research.microsoft.com/hlabinding/> | Adaptive Double Threading. 3D structures-based model (binding energy predictor). | I |
| **HLArestrictor 1.2** | <http://www.cbs.dtu.dk/services/HLArestrictor/> | Based on % rank score | I |
| **IEDB Class I Immunogenicity** | <http://tools.iedb.org/immunogenicity/> | This tool predicts the immunogenicity of MHC I (pMHC) complex using the amino acid properties and the position within the peptide. | I |
| **IEDB MHC-I Binding Predictions** | <http://tools.iedb.org/mhci/> | Framework including ANN, SMM, SMMPMBEC, Comblib, Consensus, NetMHCpan, NetMHCcons and PickPocket. | I |
| **IEDB MHC-I processing predictions** | <http://tools.iedb.org/processing/> | Framework including ANN, SMM, SMMPMBEC, Comblib, Consensus, NetMHCpan, NetMHCcons and PickPocket. | I |
| **IEDB MHC-NP** | <http://tools.iedb.org/mhcnp/help/> | Predict binding/nonbinding peptides for a given allele and predict naturally processed peptides. | I |
| **KISS** | <http://cbio.ensmp.fr/kiss/> | SVM using a multitask kernel | I |
| **LpPep** | <http://zlab.bu.edu/zhiping/lppep.html> | Linear programming (jack-knife procedure) | I |
| **MAPPP** | <http://www.mpiib-berlin.mpg.de/MAPPP/binding.html> | Based on a score calculated by BIMAS and SYFPEITHI for each subsequence. QM and Motifs | I |
| **MMBPred** | <http://crdd.osdd.net/raghava/mmbpred/> | QM | I |
| **NetChop 3.1** | <http://www.cbs.dtu.dk/services/NetChop/> | ANN | I |
| **NetCTL 1.2** | <http://www.cbs.dtu.dk/services/NetCTL/> | ANN | I |
| **NetCTLpan 1.1** | <http://www.cbs.dtu.dk/services/NetCTLpan/> | ANN | I |
| **NetMHC 4.0** | <http://www.cbs.dtu.dk/services/NetMHC> | ANN and weighted matrices | I |
| **NetMHCcons 1.1** | <http://www.cbs.dtu.dk/services/NetMHCcons/> | Integrating three methods: NetMHC (ANN), NetMHCpan (Pan specific ANN) and PickPocket (Matrix based) | I |
| **NetMHCpan 2.8** | <http://www.cbs.dtu.dk/services/NetMHCpan/> | Pan specific ANN | I |
| **NetMHCstab 1.0** | <http://www.cbs.dtu.dk/services/NetMHCstab-1.0/> | ANN | I |
| **NetTepi 1.0** | <http://www.cbs.dtu.dk/services/NetTepi/> | Integrates three methods: peptide-MHC binding affinity using NetMHCcons, peptide-MHC stability using the NetMHCstab and T-cell propensity. | I |
| **Pcleavage** | <http://crdd.osdd.net/raghava/pcleavage/> | SVM | I |
| **PEPVAC** | <http://bio.dfci.harvard.edu/PEPVAC/> | PSSM Profile-matrices | I |
| **PREDEP** | <http://margalit.huji.ac.il/Teppred/mhc-bind/index.html> | Threading and Motif. | I |
| **PREDmafa** | <http://cvc.dfci.harvard.edu/mafa/> | QM | I |
| **ProPred1** | <http://www.imtech.res.in/raghava/propred1> | Based on QM, proteasomal cleavage and promiscuous peptides. | I |
| **SliDER** | http:// modlab-cadd.ethz.ch/software/slider/ | ANN and SVM | I |
| **SMM** | <http://zlab.bu.edu/SMM/> | linear programming (SMM) | I |
| **SVMHC** | <http://www-bs.informatik.unituebingen.de/Services/SVMHC>  <http://svrmhc.biolead.org/> | SVM | I |
| **TAPpred** | <http://crdd.osdd.net/raghava/tappred/> | SVM | I |
| **T-EPITOPE Designer** | <http://www.bioinformation.net/ted/index.html> | Structure modelling (Virtual pockets in 3D). | I |
| **WAPP** | <http://abi.inf.uni-tuebingen.de/Services/WAPP/information> | SVMHC | I |
| **IFNepitope** | <http://crdd.osdd.net/raghava/ifnepitope/> | Motif based SVM, and SVM hybrid. | IFN-gamma inducing epitope |
| **EpiDOCK** | <http://epidock.ddg-pharmfac.net/> | Structure molecular docking | II |
| **EpiTOP** | <http://www.pharmfac.net/EpiTOP/> | Proteochemometrics (QSAR) QM | II |
| **FDR4** | <http://crdd.osdd.net/raghava/fdr4/> | SVMOT | II |
| **HLA-DR4Pred** | <http://www.imtech.res.in/raghava/hladr4pred/> | SVM and ANN | II |
| **IEDB MHC-II Binding Predictions** | <http://tools.iedb.org/mhcii/> | Consensus, CombLib, NN-align (netMHCII-2.2), SMM-align (netMHCII-1.1), Sturniolo, and NetMHCIIpan. | II |
| **IL4pred** | <http://crdd.osdd.net/raghava/il4pred/> | SVM, Merci Motif based, Hybrid (SVM + Motif) based, and Swissprot based. | II |
| **MetaMHCIIPan** | <http://www.biokdd.fudan.edu.cn/Service/MetaMHC.html> | A pan-specific consensus | II |
| **MHC** | <http://crdd.osdd.net/raghava/mhc/> | Motifs Matrix Optimization Technique | II |
| **MHC2MIL** | <http://datamining-iip.fudan.edu.cn/service/MHC2MIL/index.html> | Pan-specific: PSSM, ANN, kernel based methods, and multiple instance learning based methods. | II |
| **MHC2Pred** | <http://crdd.osdd.net/raghava/mhc2pred/> | SVM | II |
| **MHC2SKpan-1.0.** | <http://datamining-iip.fudan.edu.cn/service/MHC2SKpan/index.html> | MHC-II String Kernel | II |
| **MHCIIMulti** | <http://etk.informatik.uni-tuebingen.de/acl_users/credentials_cookie_auth/require_login?came_from=http%3A//etk.informatik.uni-tuebingen.de/mhciimulti/index_html/ddocument_view> | Machine learning method | II |
| **MHCMIR 1.0** | <http://ailab.ist.psu.edu/mhcmir/> | Multiple instance regression | II |
| **MHC-Thread** | <http://www.csd.abdn.ac.uk/~gjlk/MHC-Thread/> | Structure peptide threading | II |
| **NetMHCII 2.2** | <http://www.cbs.dtu.dk/services/NetMHCII/> | ANN | II |
| **NetMHCIIpan 3.1** | <http://www.cbs.dtu.dk/services/NetMHCIIpan/> | ANN | II |
| **NNAlign** | <http://www.cbs.dtu.dk/services/NNAlign/> | ANN | II |
| **PREDIVAC** | <http://predivac.biosci.uq.edu.au/> | PSSM | II |
| **ProPred** | <http://crdd.osdd.net/raghava/propred/> | QM | II |
| **RTA** | http://​bordnerlab.​org/​RTA/​ | Regularized Thermodynamic Average | II |
| **TEPITOPEpan** | <http://datamining-iip.fudan.edu.cn/service/TEPITOPEpan/TEPITOPEpan.html> | PSSM | II |
| **EpDis** | <http://bioinfo.matf.bg.ac.rs/home/downloads.wafl?cat=Software>. | NA | I and II |
| **EpiMatrix** | <http://www.epivax.com/> | Matrix-based and pocket Profile | I and II |
| **EpiToolKit 2.0** | <http://epitoolkit.de/> | Syfpeithi, BIMAS, SVMHC, SMM, SMMPMBEC, UniTope, NetMHC, NetMHCpan, NetMHCII, NetMHCIIpan, TEPITOPEpan. | I and II |
| **Epitopemap** | <http://dmnfarrell.github.io/epitopemap/> | The Tepitope method is the Python implementation of TEPITOPEPan, NetMHCIIpan and IEDB MHCI | I and II |
| **FRED** | <http://abi.inf.uni-tuebingen.de/Software/FRED/index.html> | Machine learning method | I and II |
| **HLAPRED** | <http://crdd.osdd.net/raghava/hlapred/> | QM | I and II |
| **Hotspot Hunter** | <http://antigen.i2r.a-star.edu.sg/hh/> | ANN and SVM | I and II |
| **MetaMHC** | <http://www.biokdd.fudan.edu.cn/Service/MetaMHC.html> | Local Alignment (LA) kernel | I and II |
| **MHCBench** | <http://crdd.osdd.net/raghava/mhcbench/> | Evaluate and compare the performance of the old/new prediction methods in terms of the threshold dependent and independent parameters | I and II |
| **MHC-BPS** | <http://bidd.cz3.nus.edu.sg/mhc/> | SVM | I and II |
| **MHCcluster 2.0** | <http://www.cbs.dtu.dk/services/MHCcluster/> | Based on predicted binding specificity.  MHC class I peptide binding predictions using NetMHCpan, and MHC class II peptide binding predictions using NetMHCIIpan | I and II |
| **MHCpred** | <http://www.ddg-pharmfac.net/mhcpred/MHCPred/> | QSAR | I and II |
| **MOTIF SCAN** | [http://www.hiv.lanl.gov/content/immunology/motif scan/](http://www.hiv.lanl.gov/content/immunology/motif%20scan/) | Sequence Motifs | I and II |
| **MULTIPRED2** | <http://cvc.dfci.harvard.edu/multipred2/HTML/prediction2.php> | netMHCpan and netMHCIIpan (ANN and HMM) | I and II |
| **nHLApred** | <http://crdd.osdd.net/raghava/nhlapred/comp.html> | ANN and QM | I and II |
| **OptiTope** | <http://etk.informatik.uni-tuebingen.de/optitope> | Designing epitope-based vaccines by assessing the immunogenicity | I and II |
| **PepCrawler** | <http://bioinfo3d.cs.tau.ac.il/PepCrawler/about.html> | Docking and structure modelling | I and II |
| **PickPocket 1.1** | <http://www.cbs.dtu.dk/services/PickPocket/> | Position specific weight matrix (SMM) | I and II |
| **POPI2.0** | <http://iclab.life.nctu.edu.tw/POPI/> | SVM | I and II |
| **POPI2.0** | <http://iclab.life.nctu.edu.tw/POPI/> | QM | I and II |
| **PREDBALB/c** | <http://cvc.dfci.harvard.edu/balbc/> | QM | I and II |
| **Predict** | <http://research.i2r.a-star.edu.sg/fimm> | ANN | I and II |
| **RANKPEP** | <http://imed.med.ucm.es/Tools/rankpep_help.html> | PSSM or profile | I and II |
| **SNEPv2** | <http://etk.informatik.uni-tuebingen.de/snep> | SYFPEITHI, Bimas/HLA_Bind, Epidemix, SVMHC, UniTope, TEPITOPE, MHCIIMulti | I and II |
| **SVMHC** | <http://abi.inf.uni-tuebingen.de/Services/SVMHC> | SVM | I and II |
| **SVRMHC** | <http://c1.accurascience.com/SVRMHCdb/> | SVR | I and II |
| **SYFPEITHI** | <http://www.syfpeithi.de/> | MM | I and II |
| **TEpredict** | <http://tepredict.sourceforge.net/index.html> | QM | I and II |
| **TmhcPred** | <http://www.imtech.res.in/raghava/tmhcpred/index.html> | Virtual and QM | I and II |
| **Vaxign** | <http://www.violinet.org/vaxign/> | PSSM | I and II |
