# Supplementary material for: In silico design of a T-cell epitope vaccine candidate for parasitic helminth infection
Source: PLoS Pathog. 2020 Mar 23;16(3):e1008243. doi: 10.1371/journal.ppat.1008243 (PMC7117776; doi:10.1371/journal.ppat.1008243)
Supplement: S3 Table — (DOCX) [file ppat.1008243.s005.docx]

**S3 Table.** MHC-II T-cell epitopes dataset used to evaluate the MHC-II *in silico* prediction tools.

| **Organism** | **Protein** | **Epitope origin**  **residues** | **Amino acid sequence** | **Reference** |
| --- | --- | --- | --- | --- |
| ***Clostridium tetani*** | Tetanus Toxin  (TT) | 830-844 | QYIKANSKFIGITEL | (1-8) |
|  |  | 947-967 | SFWLRVPKVSASHLE  VSFWLRVPKVSASHL  TVSFWLRVPKVSASH |  |
|  |  | 634-653 | DVSTIVPYIGPALNI  VSTIVPYIGPALNIV  IVPYIGPALNIVKQG |  |
|  |  | 950–969 | NNFTVSFWLRVPKVS  TVSFWLRVPKVSASH  SFWLRVPKVSASHL  SFWLRVPKVSASHLE |  |
|  |  | 1273-1284 | GQIGNDPNRDIL |  |
| ***Corynebacterium diphtheriae*** | Diphtheria Toxin  (DTX) | 271–290 | PVFAGANYAAWAVNV  AGANYAAWAVNVAQV  GANYAAWAVNVAQVI  ANYAAWAVNVAQVID  NYAAWAVNVAQVIDS  YAAWAVNVAQVIDSE | (2, 9-11) |
|  |  | 331–350 | QSIALSSLMVAQAIP  LSSLMVAQAIPLVGE  ALSSLMVAQAIPLVG  IALSSLMVAQAIPLV  SSLMVAQAIPLVGEL  AQSIALSSLMVAQAI  SIALSSLMVAQAIPL |  |
|  |  | 321–340 | VHHNTEEIVAQSIAL  VAQSIALSSLMVAQA  IVAQSIALSSLMVAQ |  |
|  |  | 351–370 | VDIGFAAYNFVESII  DIGFAAYNFVESIIN  IGFAAYNFVESIINL  GFAAYNFVESIINLF  FAAYNFVESIINLFQ |  |
|  |  | 411–430 | QGESGHDIKITAENT  GHDIKITAENTPLPI  SGHDIKITAENTAEN  ESGHDIKITAENTPL |  |
|  |  | 431–450 | GVLLPTIPGKLDVNK  TIPGKLDVNKSKTHI  PTIPGKLDVNKSKTH |  |
| ***P. falciparum*** | Erythrocyte binding proteins (EBP3) | 12-27 | LVSEEIVTEEGSVAQE  VSEEIVTEEGSVAQE | (8, 12-16) |
|  | Circumsporozoite (CS) | 379-398  326-345 | AKMEKCSSVFNVVNS  IEKKIAKMEKCSSVF  EYLNKIQNSLSTEWS  NKIQNSLSTEWSPCS |  |
| ***Mycobacterium tuberculosis*** | 6 kDa Early secretory antigenic target (ESAT-6) | 1-20  71–90 | QQWNFAGIEAAASAI  EQQWNFAGIEAAASA  TEQQWNFAGIEAAAS  MTEQQWNFAGIEAAA  QWNFAGIEAAASAIQ  WNFAGIEAAASAIQG  NLARTISEAGQAMAS  LARTISEAGQAMAST  ARTISEAGQAMASTE  RTISEAGQAMASTEG | (17-29) |
|  | Antigen 85B (Ag85B) | 240-254  154-173  164-183  134-153144-163284-303 | FQDAYNAAGGHNAVF  SAMILAAYHPQQFIYAGSLS AYHPQQFIYAGSLSA  QQFIYAGSLSALLDP  QFIYAGSLSALLDPS  FIYAGSLSALLDPSQ  IYAGSLSALLDPSQG  ANRAVKPTGSAAIGL  NRAVKPTGSAAIGLS  RAVKPTGSAAIGLSM  AVKPTGSAAIGLSMA  AAIGLSMAGSSAMIL  AIGLSMAGSSAMILA  IGLSMAGSSAMILAA  GLSMAGSSAMILAAY  LSMAGSSAMILAAYH  THSWEYWGAQLNAMK  HSWEYWGAQLNAMKG | (21, 30-36) |
|  | 38 kDa | 350-369 | DQVHFQPLPPAVVKL  QVHFQPLPPAVVKLS  VHFQPLPPAVVKLSD  HFQPLPPAVVKLSDA  FQPLPPAVVKLSKDAS | (32, 34, 37) |
| ***Leishmania major*** | Surface proteinase of 63 kDa (GP63) | 1-13  48-61  154-168  158-173  378-393  385-401  394-409  95-130  146-171 | VRDVNWGALRIAVS  LTNEKRDILVKHLIP  YDQLVTRVVTHEMAH  TRVVTHEMAHALGFS  PFNVFSDAARCIDGA  AARCIDGAFRPKATD  RPKATDGIVKSYAGL  DFVMYVASVPSEEGV  PAANIASRYDQLVTR | (38-41) |
|  | *Leishmania* homologue of mammalian RACKs, the receptors for activated C kinase (LACK) | 156-173  161-173 | ICFSPSLEHPIVVSG SLEHPIVVSGSWD  EHPIVVSGSWDNT | (42-46) |
| ***Leishmania infantum*** | Cysteine peptidase A (CPA) | 149-163  246-260  114-128  257-271  32-46  312-326 | MCGSCWAFATTGNIE  PHDEEEIAAYVGKNG  KDYKEHVHVDDSVRS  GKNGPVAVAVDATTW  GVDDFIASAHYGRFK  GSSWGEKGYIRLAMG | (47, 48) |
|  | Histone 1 like protein (H1) | 1-15  27-41  32-46  52-56  2-16  26-40  31-45  29-43  30-44  25-39  24-38  28-42  5-19  4-18  48-62  33-47  49-63  35-49 | MSSDSAVAALSAAMT  KTAAKKAAAKKAAAK  KAAAKKAAAKKAGAK  KAAAKKAAAKKAGAK  SSDSAVAALSAAMTS  KKTAAKKAAAKKAAA  KKAAAKKAAAKKAGA  AAKKAAAKKAAAKKA  AKKAAAKKAAAKKAG  PKKTAAKKAAAKKAA  SPKKTAAKKAAAKKA  TAAKKAAAKKAAAKK  SAVAALSAAMTSPQK  DSAVAALSAAMTSPQ  AGAKKAVRKVATPKK  AAAKKAAAKKAGAKK  GAKKAVRKVATPKKP  AKKAAAKKAGAKKAG  AAKKAAAKKAGAKKA |  |
|  | *Leishmania* eukaryotic initiation factor (LeIF) | 100-114  166-180  140-154  102-116  103-117  101-115  139-153  99-113  98-112  141-155  49-63  48-62  50-64  97-111 | LSPTRELALQTAEVI  ALRTESLRVLVLDEA  DLRKLQAGVIVAVGT  PTRELALQTAEVISR  TRELALQTAEVISRI  SPTRELALQTAEVIS  DDLRKLQAGVIVAVG  VLSPTRELALQTAEV  LVLSPTRELALQTAE  LRKLQAGVIVAVGTP  PSSIQQRAIAPFTRG  KPSSIQQRAIAPFTR  SSIQQRAIAPFTRGG  GLVLSPTRELALQTA  SNHTVSSMHAEMPKS |  |
| ***H. pylori*** | Urease subunit beta | 321-339  327–334 | CHHLDKSIKEDVQFADSRI  SIKEDVQFADSRIRP | (49, 50) |
| ***Schistosoma mansoni*** | Major egg antigen p40 | 234-246 | PKSDNQIKAVPASQA | (51-54) |
| ***Salmonella typhimurium*** | Flagellar filament protein (FliC) | 427-441  428-442 | GAVQNRFNSAITNLG  AVQNRFNSAITNLGN | (55-57) |
| ***T. muris*** | Proteasome subunit beta | 1-15  2-16  3-17  4-18  24-38  25-39  26-40  27-41 | METYPVSRVNPAKIS  ETYPVSRVNPAKISQ  TYPVSRVNPAKISQS  YPVSRVNPAKISQSP  SVVALMFDGGVVIAA  VVALMFDGGVVIAAD  VALMFDGGVVIAADT  ALMFDGGVVIAADTM  LMFDGGVVIAADTML | NA |
| **Hen egg-white lysozyme** | Ovalbumin | 323-339 | ISQAVHAAHAEINEAG  SQAVHAAHAEINEAGR | (58, 59) |
