# Supplementary material for: In silico design of a T-cell epitope vaccine candidate for parasitic helminth infection
Source: PLoS Pathog. 2020 Mar 23;16(3):e1008243. doi: 10.1371/journal.ppat.1008243 (PMC7117776; doi:10.1371/journal.ppat.1008243)
Supplement: S4 Table — (DOCX) [file ppat.1008243.s006.docx]

**S4 Table.** List of MHC-II T-cell epitope sequences and primers used to amplify the target.

| **Protein**  **(Uniprot ID number)** | **Epitope name**  **amino acid sequence** | **Primers sequence** |
| --- | --- | --- |
| **Chitin-binding domain containing protein**  **(A0A0N5E6C6)** | CBD_1243-1259_  PAGVVYQCTMPRYTLCV | Forward BamHI primer:  GATCCaacctgccggcgggcgtggtgtatcagtgcaccatgccgcgctataccctgtgcG |
|  |  | Reverse EcoRI primer:  AATTCgcacagggtatagcgcggcatggtgcactgatacaccacgcccgccggcaggttG |
| **Chitin-binding domain containing protein**  **(A0A0N5DK22)** | CBD_241-257_  GRTTPVTSAPTTVTTER | Forward BamHI primer:  GATCCtgcaccggccgcaccaccccggtgaccagcgcgccgaccaccgtgaccaccgaacgcagcccgG |
|  |  | Reverse EcoRI primer:  AATTCcgggctgcgttcggtggtcacggtggtcggcgcgctggtcaccggggtggtgcggccggtgcaG |
| **Chymotrypsin-like serine proteases**  **(A0A0N5DUC1)** | CLSP_143-158_  AILQLAKPVPFSNTVR | Forward BamHI primer:  GATCCggcgtggcgattctgcagctggcgaaaccggtgccgtttagcaacaccgtgcgcccgattG |
|  |  | Reverse EcoRI primer:  AATTCaatcgggcgcacggtgttgctaaacggcaccggtttcgccagctgcagaatcgccacgccG |
| **Chymotrypsin-like serine proteases**  **(A0A0N5DUC1)** | CLSP_398-416_  SDHQEGYPVSPSVHIVSLA | Forward BamHI primer:  GATCCcatgtgagcgatcatcaggaaggctatccggtgagcccgagcgtgcatattgtgagcctggcggaaggcG |
|  |  | Reverse EcoRI primer:  AATTCgccttccgccaggctcacaatatgcacgctcgggctcaccggatagccttcctgatgatcgctcacatgG |

The restriction sites and the two extra amino acids bases are colored in green and red, respectively.
